# Thermodynamic Evaluation of Novel 1,2,4-Triazolium Alanine Ionic Liquids as Sustainable Heat-Transfer Media

**DOI:** 10.3390/molecules29225227

**Published:** 2024-11-05

**Authors:** Kunhao Liang, Haiyun Yao, Jing Qiao, Shan Gao, Mingji Zong, Fengshou Liu, Qili Yang, Lanju Liang, Dawei Fang

**Affiliations:** 1 School of Opto-Electronic Engineering, Zaozhuang University, Zaozhuang 277160, China; 18940180325@163.com (K.L.); haiyun1990yao@163.com (H.Y.); 18804069795@163.com (J.Q.); smilegs@126.com (S.G.); zongmj@163.com (M.Z.); fengshouliu@126.com (F.L.); hanbaomami@163.com (Q.Y.); 2 Institute of Rare and Scattered Elements, College of Chemistry, Liaoning University, Shenyang 110036, China

**Keywords:** 1-ethyl-4-alkyl-1,2,4-triazolium, alanine, ionic liquid, thermophysical parameter, heat-transfer fluid

## Abstract

Ionic liquids, which are widely recognized as environmentally friendly solvents, stand out as promising alternatives to traditional heat-transfer fluids due to their outstanding heat-storage and heat-transfer capabilities. In the course of our ongoing research, we successfully synthesized ionic liquids 1-ethyl-4-alkyl-1,2,4-triazolium alanine [Taz(2,*n*)][Ala], where (*n* = 4, 5); in this study, we present comprehensive data on their density, surface tension, isobaric molar heat capacity, and thermal conductivity for the first time. The key thermophysical parameters influencing the heat-transfer process, such as thermal expansibility, compressibility, isochoric heat capacity, and heat-storage density, were meticulously calculated from experimental data. Upon comparison with previously reported ionic liquids and commercially utilized heat-transfer fluids, [Taz(2,*n*)][Ala] demonstrated superior heat-storage and heat-transfer performance, particularly in terms of heat-storage density (~2.63 MJ·m^−3^·K^−1^), thermal conductivity (~0.190 W·m^−1^·K^−1^), and melting temperature (~226 K). Additionally, the presence of the alanine anion in [Taz(2,*n*)][Ala] provides more possibilities for its functional application.

## 1. Introduction

The growing emphasis on environmental conservation and energy optimization has significantly advanced the applications of fluid processes in contemporary chemistry and process engineering, especially within the realm of heat-transfer engineering [1]. In recent years, the pressing need to enhance thermal processes and improve heat-transfer efficiencies has been consistently highlighted. For processes requiring the transfer of thermal energy between different systems, heat-transfer fluids (HTFs) have emerged as a pivotal factor influencing heat-transfer performance [2]. Generally, HTFs must possess favorable thermophysical characteristics, including high thermal stability, excellent fluidity, low vapor pressure, and outstanding thermal conductivity [3]. Prior research has underscored the critical role of thermophysical properties in determining design parameters and equipment performance across various applications, such as heat distillation columns, exchangers, and reactors [4,5,6,7].

Organic solvents, which are widely employed as HTFs, inherently possess limitations such as high vapor pressures and susceptibility to leaks, indirectly increasing their safety risks and maintenance costs [1,3,7,8,9,10,11]. Whether driven by performance or safety considerations, the distinctive attributes of ionic liquids (ILs), including lower vapor pressure, a broader liquid range, enhanced thermal stability, and non-flammability, position them as viable alternatives to traditional HTFs [12,13,14,15]. França et al. delved into the impact of thermophysical properties on the chemical process design of a shell-and-tube heat exchanger, exploring the feasibility of ILs, specifically [C*_n_*mim][BF_4_], as HTFs [1,7]. Meanwhile, Bioucas et al. presented findings on the thermophysical and toxicological properties of [C_2_mim][CH_3_SO_3_], identifying it as a promising new HTF [16]. Huminic et al. examined the effects of [C_4_mim][BF_4_], [C_4_mim][NTf_2_] and their ionanofluids (INFs) on the thermal performance of a flat-plate solar collector (FPSC) in a comparative study, emphasizing that ILs are good alternatives to conventional fluids in this context [17]. Singha et al. reviewed recent research into the thermophysical properties of INFs, highlighting the challenges and opportunities associated with using INFs as HTFs [18].

Expanding upon earlier research endeavors [19,20], this study is dedicated to advancing the development of innovative 1-ethyl-4-alkyl-1,2,4-triazolium alanine ILs. Our analysis primarily focuses on their crucial thermodynamic properties, aiming to augment the potential of using ILs as sustainable HTFs. Meanwhile, the difference in thermal stability between 1,2,4-triazolium ILs and analogous imidazolium ILs was investigated via a comparison with the [C*_n_*mim][Ala] ILs reported in previous work [21]. The findings presented in this study highlight that the [Taz(2,*n*)][Ala] (*n* = 4, 5) ILs exhibit substantial thermodynamic attributes, positioning them as viable candidates to meet application requirements across various fields, especially in terms of the capacity of HTFs.

## 2. Results and Discussion

### 2.1. Density and Surface Tension

**Figure 1:**
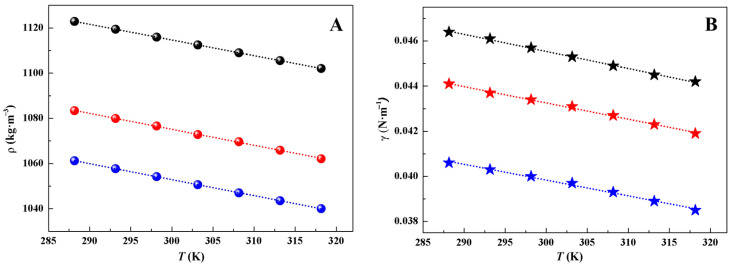
The density (**A**) and surface tension (**B**) of ILs [Taz(2,4)][Acala] (black) [19], [Taz(2,4)][Ala] (red), and [Taz(2,5)][Ala] (blue) at *T* = (288.15–318.15) K.

The correlation between the *ρ* and *γ* values of [Taz(2,*n*)][Ala] (*n* = 4, 5) with varying water contents (*w*_2_) is presented in Tables S1 and S2. The linear intercept of *ρ* or *γ* with *w*_2_ at a specific temperature is considered to be the experimental value of anhydrous ILs. The results are presented in Table 1 and visualized in Figure 1.

According to Figure 1, an increase in temperature leads to reductions in *ρ* and *γ* values, as well as a decrease in the presence of methylene (-CH_2_-) in the cation. Conversely, the introduction of acetyl (CH_3_CO-) in the anion results in the elevation of both *ρ* and *γ* [19]. These change trends are consistent with those reported in Marcinkowski’s [22] studies.

Generally, the polynomial functions used to express the relationship between *ρ* or *γ* and *T* [18] are shown in Equations (1) and (2):

(1) $$\rho \;(\mathrm{kg}\cdot m^{-3})=\sum_{i=0}^{1}a_{i}T^{i}(K)$$

(2) $$\gamma \;(N\cdot m^{-1})=\sum_{i=0}^{2}b_{i}T^{i}(K)$$

The obtained coefficients, along with the root-mean-square deviations of the fits, are presented in Table S3.

### 2.2. Isobaric Molar Heat Capacity

**Figure 2:**
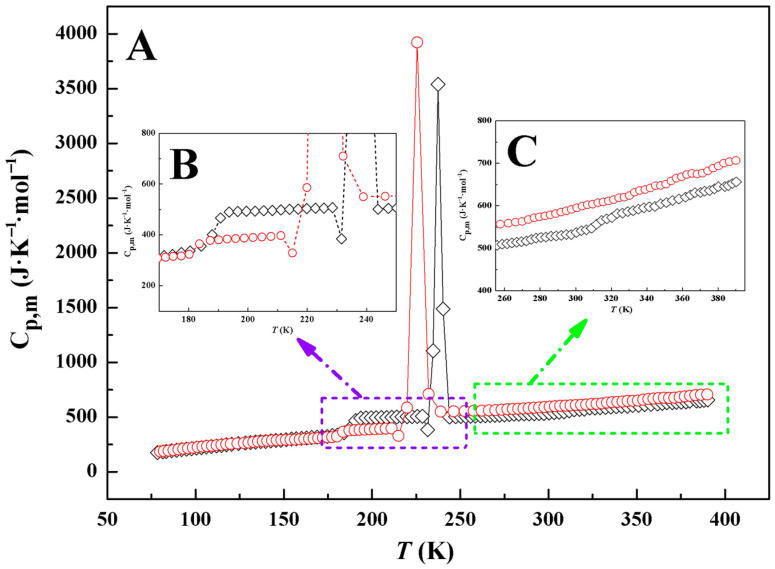
The isobaric heat capacities of 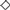 [Taz(2,4)][Ala] and 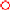 [Taz(2,5)][Ala] at (**A**) *T* = (78–390) K, (**B**) *T* = (160–250) K, and (**C**) *T* = (260–390) K.

The *C*_p,m_ values of [Taz(2,*n*)][Ala] (*n* = 4, 5) were experimentally determined across a temperature range of 78 K to 390 K, with intervals of 3 K. The obtained results are presented in Tables S4 and S5 and visually depicted in Figure 2.

Figure 2A reveals a slight increase in the *C*_p,m_ of [Taz(2,5)][Ala] compared to [Taz(2,4)][Ala] within the investigated temperature range. This phenomenon can be attributed to the larger lattice energy induced by longer alkyl chains in [Taz(2,5)][Ala] [23]. As shown in Figure 2B, both ILs exhibit curves featuring a stepped jump (~185 K) and a narrow peak (~230 K), corresponding to the glass transition temperature (*T*_g_) and melting temperature (*T*_m_), respectively (see Table 2). Notably, the curve shows a slight dip before melting, indicating a cold-crystallization process that may not always be observed in *C*_p,m_ measurements due to the discontinuity of the measured temperature [20]. An analysis of Figure 2C demonstrates the absence of any phase alteration, association, or thermal decomposition within the liquid temperature range, indicating the structural stability of the ILs.

Similarly, the *C*_p,m_ values were determined by employing a least-squares fitting approach within the temperature range of (238–390) K in the liquid phase [24].

(3) $$C_{p,m}(J\cdot K^{-1}\cdot {\mathrm{mol}}^{-1})=\sum_{i=0}^{3}c_{i}T^{i}(K)$$

The fitting results and their uncertainties are listed in Table S3.

The molar enthalpy (Δ_fus_*H*_m_) and entropy (Δ_fus_*S*_m_) of fusion can be determined by Equations (4) and (5) [25], respectively; the results are also presented in Table 2.

(4) $$\Delta_{\mathrm{fus}}H_{m}=(Q-n\int_{Ti}^{Tm}C_{p,m(S)}dT-n\int_{Tm}^{Tf}C_{p,m(L)}dT-\int_{Ti}^{Tf}C_{0}dT)/n$$

(5) $$\Delta_{\mathrm{fus}}S_{m}=\Delta_{\mathrm{fus}}H_{m}/T_{m}$$

From Table 2, it can be observed that [Taz(2,5)][Ala] exhibited a higher *T*_m_ value than [Taz(2,4)][Acala] [19] and [Taz(2,4)][Ala]; this disparity could potentially be attributed to the existence of the *H-π* bond in the triazolium cation. In addition, the order of Δ_fus_*H*_m_ was [Taz(2,5)][Ala] > [Taz(2,4)][Ala] > [Taz(2,4)][Acala] [19], indicating that the introduction of -CH_2_- to the cation led to an increase in Δ_fus_*H*_m_, while the inclusion of CH_3_CO- in the anion resulted in a decrease. The alterations in the behavior of Δ_fus_*S*_m_ demonstrated a comparable pattern.

### 2.3. Derived Properties

**Figure 3:**
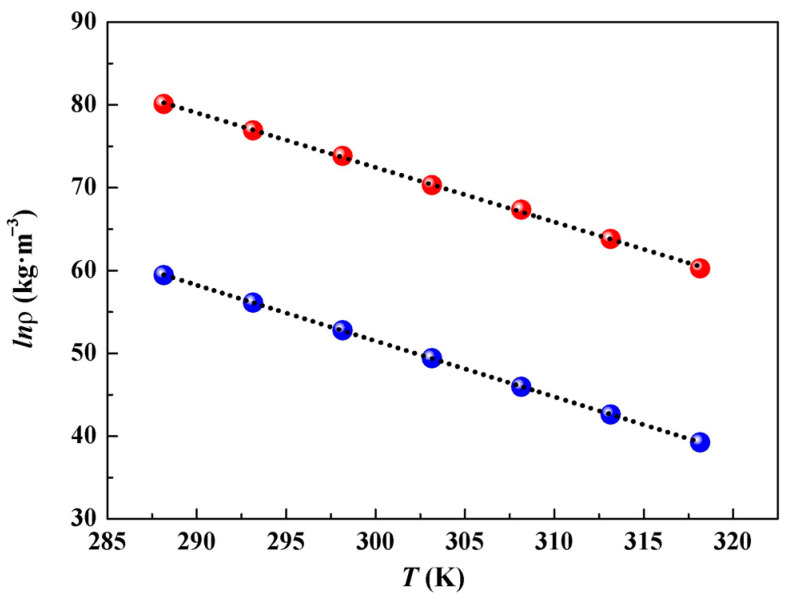
Plot of the natural logarithm of density (ln*ρ*) vs. temperature (*T*) for the ILs ([Taz(2,4)][Ala] (red): ln*ρ* = 0.2752–6.59 × 10^−4^ *T*, *r*^2^ = 0.999, *sd* = 2016 × 10^−3^; [Taz(2,5)][Ala] (blue): ln*ρ* = 0.2537–6.74 × 10^−4^ *T*, *r*^2^ = 0.999, *sd* = 4.33 × 10^−4^).

The thermal expansion coefficient (*α*_p_) can be derived using the following equation:

(6) $$\alpha_{p}=(1/V){\left(\partial V/\partial T\right)}_{P}=-{\left(\partial \ln\rho /\partial T\right)}_{P},$$

revealing a well-aligned linear relationship by plotting ln*ρ* against *T* (see Figure 3), where a negative value of the slope represents *α*_p_. The *α*_p_ values of [Taz(2,4)][Ala] (6.59 × 10^−4^ K^−1^) and [Taz(2,5)][Ala] (6.74 × 10^−4^ K^−1^) are close to those of the ILs [Ch][Ala] (4.84 × 10^−4^ K^−1^), [C*_n_*MIm][Ala] (4.92 × 10^−4^–5.81 × 10^−4^ K^−1^), and [Taz(2.4)][Acala] (6.25 × 10^−4^ K^−1^) at 298.15 K [19,26,27]. Given that the effect of temperature is almost imperceptible [9,10], *α*_p_ can be approximated as a temperature-independent fixed value.

The speed of sound (*c*) and isothermal compressibility coefficient (*κ*_T_) can be obtained by Equations (7) and (8) [28], respectively.

(7) $$c={\left[\gamma /(6.3\times 10^{-10}\rho )\right]}^{2/3}$$

(8) $$\kappa_{T}=\rho^{-1}c^{-2}+{\alpha_{p}}^{2}VTC_{p,m},$$

where *V* is the molar volume and the product of *ρ*^−1^*c*^−2^ denotes the isentropic compressibility coefficient (*κ*_S_). The values of *V*, *c*, *κ*_S_, *κ*_T_, and *C*_p,m_ are presented in Table 3.

According to Table 3, the ranking of *κ*_T_ is as follows: [Taz(2,5)][Ala] exhibits a higher value than [Taz(2,4)][Ala], which, in turn, has a higher value than [Taz(2,4)][Acala] [19]. This indicates that the introduction of a -CH_2_- group in the triazolium cation leads to an increase in *κ*_T_, while the incorporation of a CH_3_CO- group in the alanine anion results in a decrease.

Our findings suggest that the structural rigidity of the compound is enhanced by the simplification of the alkyl chain in the cation homologues of triazolium alanine ILs. The conclusion indicates that the shorter the alkyl chain of the triazolium cation in an [Taz(2,*n*)][Ala] IL, the more effectively the structural rigidity of the compound is enhanced. The values of *κ*_S_ are slightly smaller (~7.5%) than those of *κ*_T_ and also change with *T* in the same manner.

In comparison, the *κ*_T_ values of [Taz(2,*n*)][Ala] (4.98 × 10^−10^–5.84 × 10^−10^ Pa^−1^) are similar to those of [C*_n_*C_1_im]_2_[Co(NCS)_4_] (4.85 × 10^−10^–5.61 × 10^−10^ Pa^−1^) at the same temperature and pressure [8]. Therefore, the ILs studied in this work can reasonably be considered to be potential candidates for hydraulic fluids [9].

As an important and widely measurable thermodynamic quantity, the isochoric heat capacity (*C*_v,m_) can be obtained using the well known relation outlined in [29]; the results are listed in Table 3.

(9) $$C_{v,m}=C_{p,m}-{\alpha_{p}}^{2}{\kappa_{T}}^{-1}VT$$

For comparison, the order of *C*_v,m_ is [Taz(2,5)][Ala] > [Taz(2,4)][Acala] [19] > [Taz(2,4)][Ala], indicating that the introduction of -CH_2_- to the triazolium cation or CH_3_CO- in the alanine anion leads to an increase in *C*_v,m_.

### 2.4. Thermal Stability

Thermal stability plays a pivotal role in ensuring the safety and performance of industrial applications, particularly in heat-transfer processes. Table S6 provides information on the composition and operating temperatures of commonly used HTFs and ILs.

**Figure 4:**
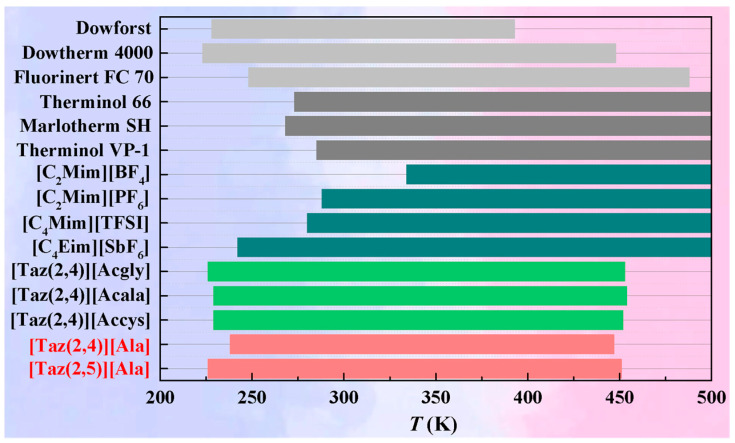
Comparison of operating temperature ranges of [Taz(2,*n*)][Ala] (*n* = 4, 5) with selected ILs ([C_2_MIm][BF_4_] [3], [C_2_MIm][PF_6_] [3], [C_4_MIm][TFSI] [11], [C_4_EIm]SbF_6_] [20], [Taz(2,4)][Acgly] [19], [Taz(2,4)][Acala] [19], and [Taz(2,4)][Accys] [19]) and with commercial HTFs (Dowforst [3], Dowtherm 4000 [3], Fluorinert FC70 [3], Therminol 66 [9], Marlotherm SH [10], and Therminol VP-1 [10]).

ILs can be securely employed within the temperature range from their *T*_m_ to the temperature of significant weight loss (<5%). The *T*_m_ values for [Taz(2,*n*)][Ala] (*n* = 4, 5), derived from the *C*_p,m_ data, are 238 K and 226 K, respectively. TG analysis revealed no significant weight losses until 447 K, demonstrating the commendable thermal stability of [Taz(2,*n*)][Ala] (*n* = 4, 5). The operating temperature ranges of [Taz(2,*n*)][Ala] (*n* = 4, 5), alongside several common ILs [8,9,10,19,20] and commercial HTFs [3,9,10], are compared and illustrated in Figure 4. It can be seen that [Taz(2,*n*)][Ala] (*n* = 4, 5) maintains its liquid state at approximately −40 °C, showcasing the ability of ILs to efficiently function in environments characterized by extremely low temperatures—an advantageous feature for HTFs. Furthermore, the upper operating temperature limits for both ILs are comparable to those of ILs like 1-ethyl-4-butyl-1,2,4-triazolium acetyl amino acid [19] and HTF Dowtherm 4000 [9].

It is worth mentioning that there seems to be debate about whether the thermal stability of 1,2,4-triazolium ILs is better than that of analogous imidazolium ILs. Compared with previous studies, it was found that the thermal stability of [C*_n_*mim)][Ala] (*n* = 4, 5) was greater than that of [Taz(2,*n*)][Ala] (*n* = 4, 5) [21]. This is consistent with Brauer’s [30] and Tokuda’s [31] studies and contrary to Chand’s [32]. The reason for this contradiction may be related to the choice of anion.

### 2.5. Heat-Storage Density

For HTFs, a higher heat-storage density (*E*) saves both volume and cost in cases of transferring the same amount of heat. As a necessary parameter for estimating heat-transfer demands, the *E* can be obtained from Equation (10):

(10) $$E=C_{p,m}/V$$

The *E* values for [Taz(2,*n*)][Ala] (*n* = 4, 5) within the temperature range of (288.15–318.15) K are summarized in Table 4. Notably, the *E* values of [Taz(2,5)][Ala] exhibit a slight increase compared to [Taz(2,4)][Ala], suggesting that the incorporation of -CH_2_- in the anion positively contributes to the enhancement of the *E* value. To facilitate a comparative analysis, the *E* values from previously reported ILs with the potential to act as HTFs [3,8,9,10,11,19,33,34], as well as commercial HTFs [8,9,10,11], were compiled at 313.15 K.

**Figure 5:**
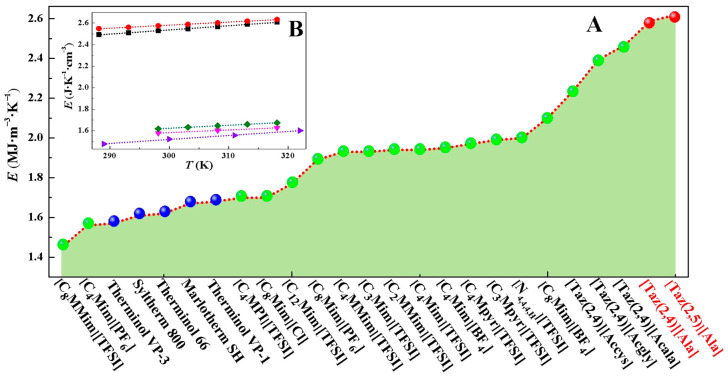
(**A**) Comparison of the heat-storage density (*E*) of [Taz(2,*n*)][Ala] (*n* = 4, 5) at 313.15 K under atmospheric pressure with the *E* of selected ILs ([C_8_MMIm][TFSI] [34], [C_4_Mim][PF_6_] [3], [C_4_MPI][TFSI] [3], [C_8_MIm][Cl] [33], [C_4_MMIm][TFSI] [10], [C_3_MIm][TFSI] [10], [C_2_MMIm][TFSI] [10], [C_4_MIm][TFSI] [10], [C_4_Mim][BF_4_] [3], [C_4_MPyr][TFSI] [9], [C_3_MPyr][TFSI] [9], [C_4_MMIm][TFSI] [10], [N_4,4,4,4_][TFSI] [33], [C_8_Mim][BF_4_] [3], [Taz(2,4)][Accys] [19], [Taz(2,4)][Acgly] [19], and [Taz(2,4)][Acala] [19]) and with commercial HTFs (Therminol VP-3 [10], Syltherm 800 [3], Therminol 66 [9], Marlotherm SH [9], and Therminol VP-1 [9]). (**B**) The effect of temperature on the *E* of 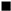 [Taz(2,4)][Ala] and 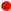 [Taz(2,5)][Ala] with that on commercial HTFs 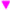 Therminol 66 [9], 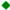 Marlotherm SH [9], and 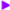 Therminol VP-3 [10].

The results indicate that the *E* (MJ·m^−3^·K^−1^) values of [Taz(2,4)][Ala] (2.588) and [Taz(2,5)][Ala] (2.618) surpass those of ILs such as [C_4_MIm][TFSI] (1.94) [9], [C_4_MMIm][TFSI] (1.93) [10], [C_4_MPyr][TFSI] (1.97) [9], and [Taz(2,4)][Acala] (2.47) [19], as well as those of commercial HTFs Therminol VP-1 (1.68) [9], Therminol 66 (1.62) [9], and Marlotherm SH (1.67) [9] (Figure 5A). The advantage in terms of *E* is more pronounced for [Taz(2,*n*)][Ala] (*n* = 4, 5) compared to the selected ILs and commercial HTFs. The excellent *E* of the target ILs may depend on the following factors: (1) N in triazolium and COO- and NH_2_- in alanine, resulting in the formation of strong H bonds between molecules; (2) the aromaticity and electron cloud distribution of the triazolium, resulting in a more complex interaction with alanine; and (3) the structural combination of triazolium and alanine, leading to higher steric hindrance. Furthermore, the *E* value of [Taz(2,*n*)][Ala] (*n* = 4, 5) is only marginally affected by temperature, as demonstrated in Figure 5B, when compared to commercial HTFs such as Therminol VP-3, Therminol 66, and Marlotherm SH within the specified range of test temperatures. In summary, it is evident that the investigated ILs exhibit superior thermal storage properties compared to commercial HTFs.

### 2.6. Thermal Conductivity

The outstanding heat-storage capabilities exhibited by [Taz(2,*n*)][Ala] (*n* = 4, 5) prompted a comprehensive investigation of their thermal conductivity (*λ*) within the context of HTFs. The experimental *λ* data for [Taz(2,*n*)][Ala] (*n* = 4, 5) are meticulously listed in Table 5 and characterized by the following polynomial function:

(11) $$\lambda \;(W\cdot m^{-1}\cdot K^{-1})=\sum_{i=0}^{1}d_{i}T^{i}(K)$$

The derived coefficients, along with the root-mean-square deviations of the fits, are presented in Table S3.

**Figure 6:**
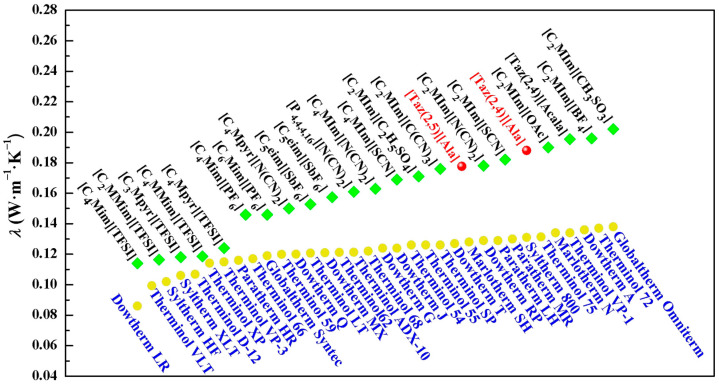
Comparison of the thermal conductivity (*λ*) of [Taz(2,*n*)][Ala] (*n* = 4, 5) with selected ILs ([C_2_MIm][N(CN)_2_] [40], [C_4_MIm][N(CN)_2_] [40], [C_4_MPyr][N(CN)_2_] [40], [C_4_MIm][TFSI] [35], [C_2_MMIm][TFSI] [33], [C_4_MMIm][TFSI] [33], [C_3_MPyr][TFSI] [3], [C_4_MPyr][TFSI] [3], [C_2_MIm][SCN] [1], [C_4_MIm][SCN] [1], [C_2_MIm][BF_4_] (323 K) [1], [C_4_MIm][PF_6_] (323 K) [26], [C_6_MIm][PF_6_] (323 K) [26], [C_2_MIm][C(CN)_3_] [1], [C_2_MIm][C_2_H_5_SO_4_] [40], [P_4,4,4.16_][N(CN)_2_] [40], [C_2_MIm][CH_3_SO_3_] [36], [C_2_MIm][OAc] [9], [Taz(2,4)][Acala] [19], [C_4_Eim][SbF_6_] [20], and [C_5_Eim][SbF_6_] [20]) and with commercial HTFs (Therminol 66 [10], Therminol VP-1 [10], Therminol VP-3 [9], Dowtherm A [1], Dowtherm G [1], Dowtherm J [1], Dowtherm MX [1], Dowtherm Q [1], Dowtherm RP [1], Dowtherm T [1], Syltherm XLT [1], Syltherm 800 [1], Syltherm HF [1], Paratherm HR [1], Paratherm MR [1], Globaltherm Omniterm [1], Globaltherm Syntec [1], Marlotherm SH [1], Therminol 54^a^, Therminol 55^a^, Therminol 59^a^, Therminol 62^a^, Therminol 68^a^, Therminol 72^a^, Therminol 75 (343.15 K)^a^, Therminol ADX-10^a^, Therminol D-12^a^, Therminol LT^a^, Therminol VLT^a^, Therminol SP^a^, Therminol XP^a^, Marlotherm LH^a^, Marlotherm N^a^, and Paratherm LR (311.15 K)^a^ [1,9,10,11]) at 313.15 K. ^a^ Obtained from the product information brochure available online and/or upon request to the supplier.

Similarly, the *λ* values of 20 ILs [1,3,7,8,9,12,19,20,35,36,37], along with those of 34 commercial HTFs [1,3,7,8,9] and the ILs investigated in this study, were compared at 313.15 K (refer to Table S7 and Figure 6). The findings reveal that [Taz(2,*n*)][Ala] (*n* = 4, 5) exhibited higher *λ* values compared to the majority of the selected ILs and all commercial HTFs. This underscores the promising potential of these compounds as sustainable HTFs. Similar to the enhancement of heat-storage density, the presence of strong interactions in triazolalanine ILs may promote heat transfer. Meanwhile, the conjugated double bonds contained in the triazolalanine ILs also make the molecular electron cloud more widely distributed and enhance electron delocalization, which is conducive to heat transfer. Furthermore, an examination of the relationship between the *λ* values and the structures of ILs showed a decrease in *λ* with the introduction of methylene. For [Taz(2,*n*)][Ala] (*n* = 4, 5), *λ* gradually decreases when temperatures increase in the range of 288.15–318.15 K, with the maximum attenuation ratio being less than 0.99%. This is notably lower than that of the selected commercial HTFs (1.1–3.6%) [9,10]. The minimal impact of temperature on *λ* for the two ILs under investigation is evident and is undoubtedly a favorable characteristic for their potential application as HTFs in the future.

**Table 5:** The values of the thermal conductivity (*λ*/W*·*m^−1^·K^−1^) of [Taz(2,*n*)][Ala] (*n* = 4, 5) at *T* = (288.15–318.15) K.

*T*/K	[Taz(2,4)][Ala]			[Taz(2,5)][Ala]		
288.15	^a^ 0.1896	^b^ 0.162	^c^ 0.192	^a^ 0.1812	^b^ 0.161	^c^ 0.189
293.15	^a^ 0.1893	^b^ 0.162	^c^ 0.191	^a^ 0.1810	^b^ 0.161	^c^ 0.188
298.15	^a^ 0.1889	^b^ 0.162	^c^ 0.190	^a^ 0.1807	^b^ 0.160	^c^ 0.188
303.15	^a^ 0.1886	^b^ 0.161	^c^ 0.190	^a^ 0.1804	^b^ 0.159	^c^ 0.187
308.15	^a^ 0.1882	^b^ 0.160	^c^ 0.189	^a^ 0.180	^b^ 0.158	^c^ 0.186
313.15	^a^ 0.1880	^b^ 0.160	^c^ 0.188	^a^ 0.1796	^b^ 0.157	^c^ 0.186
318.15	^a^ 0.1876	^b^ 0.160	^c^ 0.188	^a^ 0.1792	^b^ 0.156	^c^ 0.185

^a^ Experimental *λ* values with expanded uncertainty of *U*(*λ*) = 0.0051 J·mol^−1^·K^−1^ (0.95 level of confidence, *k* = 2); ^b^ *λ* values estimated using the model of Wu et al. [38]; ^c^ *λ* values estimated using the model of Oster et al. [39].

Given the labor-intensive and time-consuming nature of *λ* testing, establishing a simple yet accurate prediction model is paramount [37]. In this study, the modified Bridgman equation developed by Wu et al. [38] and the group contribution method proposed by Oster et al. [39] were employed to calculate *λ* (refer to Table 5), with the parameters used in the method of Oster et al. detailed in Table S8.

**Figure 7:**
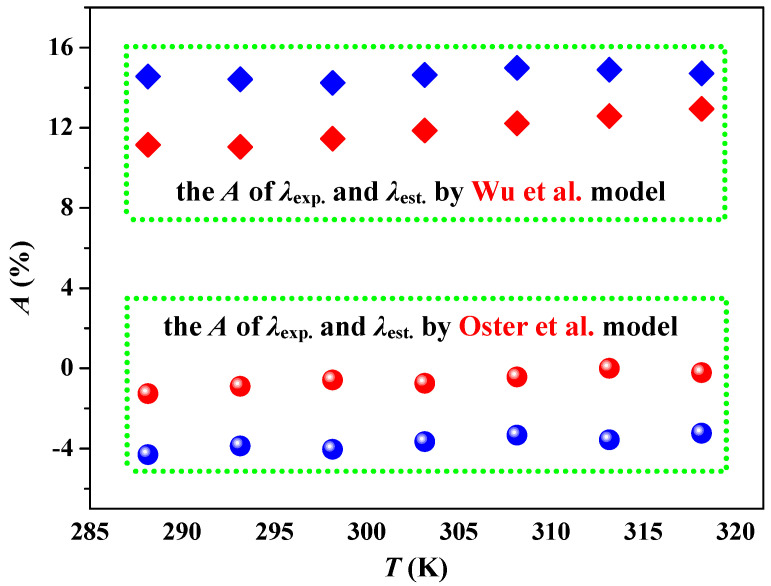
The relative deviation (*A*% = (*λ*_exp._ + *λ*_est._)/*λ*_exp._) of the experimental values (*λ*_exp._) and estimated values (*λ*_est._) [38,39] of thermal conductivity for ILs [Taz(2,4)][Ala] (blue) and [Taz(2,5)][Ala] (red).

The relative deviation of the experimental values (*λ*_exp._) and estimated values (*λ*_est._) for [Taz(2,*n*)][Ala] (*n* = 4, 5) is depicted in Figure 7. For Wu et al.’s model [38], the deviations between *λ*_exp._ and *λ*_est._ fluctuated between 11.05% (for [Taz(2,5)][Ala] at 293.15 K) and 14.98% (for [Taz(2,4)][Ala] at 308.15 K). These results highlight that Wu et al.’s model significantly underestimates the *λ* values of [Taz(2,*n*)][Ala] (*n* = 4, 5); similar underestimations were also noted in investigations by Zorębski et al. [9,10,29]. For Oster et al.’s model [39], the deviations between *λ*_exp._ and *λ*_est._ were consistently within 4.30% (for [Taz(2,5)][Ala] at 288.15 K). These results signify that the Oster model is well suited for the precise prediction of λ values for 1-ethyl-4-alkyl-1,2,4-triazolium alanine ILs. The main reasons for this difference could be that the Wu model’s samples were insufficient, among which there were almost no ionic liquids that included triazolium cations or alanine anions [38]. However, for the Oster model, based on the group contribution method, only a limited number of groups need to be considered, rather than a large number of ILs, which effectively avoids the dilemma faced when using the Wu model to predict the λ of triazolium alanine ILs [39].

## 3. Materials and Methods

### 3.1. Materials

The relevant information about the reagents used in IL synthesis is listed in Table 6.

### 3.2. Preparation and Characterization

**Figure 8:**
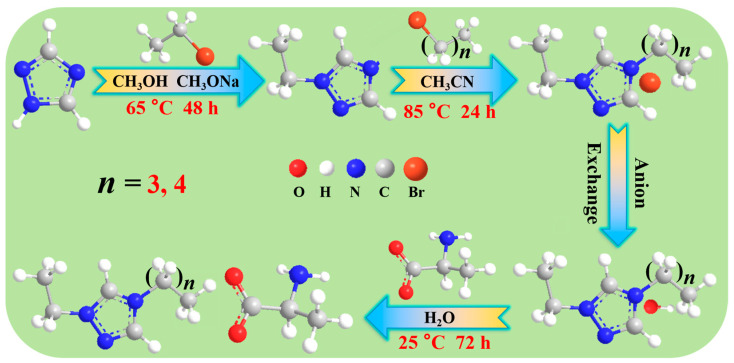
Synthesis of novel ILs [Taz(2,4)][Ala] and [Taz(2,5)][Ala].

In this study, we initially designed and synthesized [Taz(2,4)][Ala] (*M* = 242.40 g·mol^−1^) and [Taz(2,5)][Ala] (*M* = 256.42 g·mol^−1^); the complete synthetic route is illustrated in Figure 8. The process was initiated by stirring a solution of C_2_H_3_N_3_ (1,2,4-triazole), CH_3_OH, and CH_3_ONa (molar ratio = 1:1:1) in a round-bottomed flask at 25 °C; then, CH_3_CH_2_Br was added dropwise into the flask, which was stirred under reflux at 65 °C for 48 h, then distilled to obtain C_4_H_7_N_3_ (1-ethyl-1,2,4-triazole). Subsequently, the C_4_H_7_N_3_ was reacted with C*_n_*H_2*n*+1_Br (*n* = 4,5) (molar ratio = 1:1.2) to obtain [Taz(2,*n*)][Br] (*n* = 4, 5) [41]. This process was exothermic, and the reaction temperature and reaction time were 85 °C and 24 h, respectively. Then, [Taz(2,*n*)][OH] (*n* = 4,5) aqueous solutions were obtained from [Taz(2,*n*)][Br] (*n* = 4,5) using activated anion-exchange resin over a 100 cm column. Finally, the concentrations of the [Taz(2,*n*)][OH] (*n* = 4,5) aqueous solutions were titrated and reacted with the equimolar addition of *L*-Alanine at 25 °C for 72 h to obtain [Taz(2,*n*)][Ala] (*n* = 4, 5) [22,41]. The target products were evaporated under reduced pressure at 50 °C and dried in vacuo for 48 h at 55 °C. In addition, dichloromethane and acetonitrile were used as extractants in this experiment.

Furthermore, the synthesized ILs underwent characterization using ^1^H-NMR, ^13^C-NMR, and TG, as illustrated in Figures S1–S6, while their water contents were validated by a Karl Fischer moisture titrator. The structures of the [Taz(2,*n*)][Ala] (*n* = 4, 5) ILs were unequivocally confirmed, with no discernible impurity peaks detected during the spectral analysis that employed ^1^H-NMR and ^13^C-NMR techniques. Our analysis of the TG curves revealed initial decomposition temperatures of approximately 447.19 K for [Taz(2,4)][Ala] and 450.71 K for [Taz(2,5)][Ala]. The water content (with mass fraction) values were determined to be (0.0051 ± 0.0001) for [Taz(2,4)][Ala] and (0.0053 ± 0.0001) for [Taz(2,5)][Ala]. Consequently, the purity of [Taz(2,*n*)][Ala] (*n* = 4, 5) was estimated to exceed 99%.

### 3.3. Measurements of Thermodynamic Properties

Owing to the presence of hydrogen bonds between alanine ILs and water, the standard addition method (SAM) was employed to acquire the density (*ρ*) and surface tension (*γ*) of the anhydrous [Taz(2,*n*)][Ala] (*n* = 4, 5) [24]. The measurements of *ρ* and *γ* were conducted using a DMA 5000M densitometer (Anton Paar) and a DP-AW surface tension meter (Nanjing Sangli) within the temperature range of *T* = (288.15–318.15) K, respectively [37]. Prior to formal measurements, the aforementioned instruments were diligently calibrated, with the detailed specifications outlined in a preceding study [19]. The expanded uncertainty was 0.10 kg·m^−3^ for *ρ* and 0.0001 N·m^−1^ for *γ*. The molar heat capacities at constant pressure (*C*_p,m_) of [Taz(2,*n*)][Ala] (*n* = 4, 5) were determined utilizing a highly precise automated calorimeter spanning temperatures from 78 K to 390 K (see Figure S7). A thorough explanation of the calorimeter’s principle and calibration can be found in other sources [42,43,44]. The calibration data are presented in Tables S9–S13 and Figure S8 [27], with the expanded uncertainty of *C*_p,m_ set at 0.005 J·K^−1^·mol^−1^. The *λ* of [Taz(2,*n*)][Ala] (*n* = 4, 5) was assessed using a thermal constants analyzer (Hot Disk TPS 2500S, Sweden) across the temperature range of 288.15 K to 318.15 K. Prior to measurements, the instrument’s accuracy was verified with water [45]. The obtained *λ* values had an expanded uncertainty of 0.051 J·mol^−1^·K^−1^ (*k* = 2, confidence level of 0.95).

## 4. Conclusions

In this study, we successfully synthesized and characterized novel ionic liquids (ILs), denoted as [Taz(2,*n*)][Ala] (*n* = 4, 5). Our examination encompassed diverse properties, including density measurements, surface tension analysis, determination of isobaric molar heat capacity, and an exploration of thermal conductivity across varying temperatures. Utilizing the acquired experimental data, we calculated significant thermodynamic parameters such as isobaric thermal expansibility, isentropic compressibility, isothermal compressibility, isochoric heat capacity, and heat-storage density. Furthermore, we estimated the thermal conductivity of 1-ethyl-4-alkyl-1,2,4-triazolium alanine using both the group contribution method and other property-based approaches and extensively discussed the suitability of these models.

The ILs [Taz(2,*n*)][Ala] (*n* = 4, 5) analyzed in this study showcase significant potential as heat-transfer fluids (HTFs), as evidenced by comparing their heat-storage density and thermal conductivity data with those of extensively studied ILs and commonly used commercial HTFs. These ILs offer several advantages over commercial HTFs, including a higher heat-storage density (approximately 1.5 times greater) and enhanced thermal conductivity (around 1.7 times higher). Moreover, they exhibit comparable or even lower sensitivity to temperature changes compared to commercial HTFs. Additionally, the lower liquid temperature limit (~226 K) and the functional alanine anion provide a wider and more diverse range of application scenarios for ILs. Lastly, our investigation into the influence of structure on the above properties provides valuable insights for the development of future ILs with superior properties.

## Figures and Tables

**Table 1:** The density (*ρ*) and surface tension (*γ*) values of [Taz(2,*n*)][Ala] (*n* = 4, 5) at *T* = (288.15–318.15) K.

^a^*T*/K	[Taz(2,4)][Ala]		[Taz(2,5)][Ala]	
	^b^*ρ*/kg·m^−3^	10^3 c^ *γ*/N·m^−1^	^b^*ρ*/kg·m^−3^	10^3 c^ *γ*/N·m^−1^
288.15	1083.41	44.1	1061.25	40.6
293.15	1079.95	43.7	1057.72	40.3
298.15	1076.63	43.4	1054.20	40.0
303.15	1072.84	43.1	1050.66	39.7
308.15	1069.66	42.7	1047.03	39.3
313.15	1065.89	42.3	1043.54	38.9
318.15	1062.11	41.9	1040.04	38.5

The standard uncertainty (*u*) for temperature is ^a^ *u*(*T*) = 0.01 K; the expanded uncertainties with a 0.95 level of confidence (*k* = 2) for density and surface tension are ^b^ *U*(*ρ*) = 0.10 kg·m^−3^ and ^c^ *U*(*γ*) = 0.0001 N·m^−1^, respectively.

**Table 2:** Results of the phase transition of the [Taz(2,4)][Ala], [Taz(2,4)][Acala], and [Taz(2,5)][Ala] obtained from the isobaric molar heat capacity measurements.

Ionic Liquid	*T*_g_/K	*T*_m_/K	Δ_fus_*H*_m_/kJ·mol^−1^	Δ_fus_*S*_m_/J·K^−1^·mol^−1^
[Taz(2,4)][Ala]	187.898	237.350	25.872	116.200
^a^ [Taz(2,4)][Acala]	*-*	229.114	20.515	92.127
[Taz(2,5)][Ala]	183.606	225.530	27.901	126.250

^a^ Obtained in previous work [19].

**Table 3:** Values of the molar volume (*V*), thermal expansion coefficient (*α*_p_), speed of sound (*c*), isentropic compressibility coefficient (*κ*_S_), isothermal compressibility coefficient (*κ*_T_), isobaric molar heat capacities (*C*_p,m_), and isochoric molar heat capacities (*C*_v,m_) of [Taz(2,4)][Ala] and [Taz(2,5)][Ala] at *T* = (288.15–318.15) K.

*T*/K	10^4^ *V*/m^3^	10^4^ *α*_p_ /K^−1^	*c* /m·s^−1^	10^10^ *κ*_S_/Pa^−1^	10^10^ *κ*_T_/Pa^−1^	*C*_p,m_/J·mol^−1^·K^−1^	*C*_v,m_/J·mol^−1^·K^−1^
[Taz(2,4)][Ala]							
288.15	2.126	6.59	1419	4.58	4.98	530.02	488.03
293.15	2.133	6.59	1414	4.63	5.03	535.29	492.85
298.15	2.140	6.59	1407	4.69	5.09	540.89	498.17
303.15	2.147	6.59	1397	4.77	5.18	546.78	503.93
308.15	2.154	6.59	1389	4.84	5.25	552.92	509.83
313.15	2.161	6.59	1382	4.91	5.32	559.27	515.92
318.15	2.169	6.59	1374	4.98	5.40	565.78	522.19
[Taz(2,5)][Ala]							
288.15	2.281	6.74	1397	4.82	5.21	581.40	538.38
293.15	2.289	6.74	1392	4.87	5.27	586.17	542.72
298.15	2.296	6.74	1384	4.95	5.34	591.17	547.45
303.15	2.304	6.74	1371	5.06	5.46	596.38	552.73
308.15	2.312	6.74	1358	5.17	5.58	601.78	558.23
313.15	2.320	6.74	1345	5.30	5.70	607.38	563.95
318.15	2.328	6.74	1331	5.42	5.84	613.14	569.87

**Table 4:** The values of the heat-storage density (*E*/MJ·m^−3^·K^−1^) of [Taz(2,4)][Ala], [Taz(2,4)][Acala], and [Taz(2,5)][Ala] at *T* = (288.15–323.15) K.

Ionic Liquids	288.15/K	293.15/K	298.15/K	303.15/K	308.15/K	313.15/K	318.15/K
[Taz(2,4)][Ala]	2.493	2.509	2.528	2.546	2.567	2.588	2.609
^a^ [Taz(2,4)][Acala]	2.399	2.410	2.416	2.439	2.462	2.465	2.473
[Taz(2,5)][Ala]	2.549	2.561	2.575	2.588	2.603	2.618	2.634

^a^ Obtained in previous work [19].

**Table 6:** The CAS number, mass fraction purity, source, and analysis method of the reagents.

Reagent	CAS Number	Mass Fraction Purity	Source	Analysis Method
Bromoethane	74-96-4	99%	Shanghai Macklin Biochemical Co., Ltd. (Shanghai, China)	-
Bromobutane	190-65-9	99%	Shanghai Macklin Biochemical Co., Ltd. (Shanghai, China)	-
Bromopentane	110-53-2	99%	Shanghai Macklin Biochemical Co., Ltd. (Shanghai, China)	-
1,2,4-Triazole	288-88-0	99%	Shanghai Macklin Biochemical Co., Ltd. (Shanghai, China)	-
*L*-Alanine	56-41-7	99%	Shanghai Macklin Biochemical Co., Ltd. (Shanghai, China)	-
Methanol	67-56-1	99%	Tianjin Fuyu Chemical Co., Ltd. (Tianjin, China)	-
Sodium Methoxide	124-41-4	99%	Shanghai Macklin Biochemical Co., Ltd. (Shanghai, China)	-
Acetonitrile	75-05-8	99%	Tianjin Fuyu Chemical Co., Ltd. (Tianjin, China)	-
Dichloromethane	75-09-2	99%	Tianjin Fuyu Chemical Co., Ltd. (Tianjin, China)	-
α-Al_2_O_3_ (s)	1344-28-1	>99.95%	National Institute of Standards and Technology (Gaithersburg, MD, USA)	-
[Taz(2,4)][Ala]	-	>99%	Filtration, distillation, recrystallization and vacuum drying	^1^H-NMR, ^13^C-NMR, and TG
[Taz(2,5)][Ala]	-	>99%	Filtration, distillation, recrystallization and vacuum drying	^1^H-NMR, ^13^C-NMR, and TG

## Data Availability

The data presented in this study are available in the Supplementary Materials.

## Supplementary Materials

The following supporting information can be downloaded at https://www.mdpi.com/article/10.3390/molecules29225227/s1, Figure S1: ^1^H NMR spectroscopy of [Taz(2,4)][Ala] in DMSO; Figure S2: ^1^H NMR spectroscopy of [Taz(2,5)][Ala] in DMSO; Figure S3: ^13^C NMR spectroscopy of [Taz(2,4)][Ala] in DMSO; Figure S4: ^13^C NMR spectroscopy of [Taz(2,5)][Ala] in DMSO; Figure S5: Thermogravimetry of [Taz(2,4)][Ala]; Figure S6: Thermogravimetry of [Taz(2,5)][Ala]; Figure S7: Cross-sectional diagram of adiabatic calorimetric cryostat; Figure S8: The heat capacities of empty equivalent (A) and α-Al_2_O_3_ (B) in the temperature range of 78 to 400 K; Table S1: Density values (*ρ*, kg·cm^−3^) of [Taz(2,4)][Ala] and [Taz(2,5)][Ala] containing various mass fractions of water (*w*_2_) at *T* = (288.15–318.15) K; Table S2: Surface tension values (*γ*, N·m^−1^) of [Taz(2,4)][Ala] and [Taz(2,5)][Ala] containing various mass fractions of water (*w*_2_) at *T* = (288.15–318.15) K; Table S3: The fitting coefficients of the thermophysical properties dependent on temperature for [Taz(2,4)][Ala] and [Taz(2,5)][Ala]; Table S4: The experimental molar heat capacities of [Taz(2,4)][Ala] in the temperature range of 78 to 390 K; Table S5: The experimental molar heat capacities of [Taz(2,5)][Ala] in the temperature range of 79 to 400 K; Table S6: The composition and operating temperature for the most frequently used HTFs and ILs [3,8,9,10,11,19,20,33]; Table S7: The thermal conductivity for [Taz(2,4)][Ala], [Taz(2,5)][Ala], the most frequently used HTFs, and the most widely studied ILs at 313.15 K [1,3,9,19,20,26,33,35,36,40]; Table S8: Values of the boiling temperature (*T*_b_), critical temperature (*T*_c_), and important parameters for the group contribution method proposed by Oster et al.; Table S9: The first (series 1), the second (series 2) and the third (series 3) experimental heat capacities of the empty equivalent in the temperature range of 78 to 400 K; Table S10: The heat capacities of *α*-Al_2_O_3_ in the temperature range of 78 to 400 K; Table S11: The first experimental data, fitted values, and relative deviation of the empty equivalent in the temperature range of 78 to 400 K; Table S12: The heat capacities of *α*-Al_2_O_3_ in the temperature range of 79 to 400 K; Table S13: Experimental data, recommended NIST values, and relative deviation of α-Al_2_O_3_ in the temperature range of 80 to 400 K.
