# Supplementary material for: Thermodynamic Evaluation of Novel 1,2,4-Triazolium Alanine Ionic Liquids as Sustainable Heat-Transfer Media
Source: Molecules. 2024 Nov 5;29(22):5227. doi: 10.3390/molecules29225227 (PMC11596503; doi:10.3390/molecules29225227)
Supplement: Supplementary file 1 [file molecules-29-05227-s001.zip › molecules-3237963-supplementary.pdf]

Supporting Information

Thermodynamic Evaluation of Novel 1,2,4-Triazolium Alanine Ionic Liquids as Sustainable Heat-Transfer Media

Kunhao Liang [1,2], Haiyun Yao [1], Jing Qiao [1,2], Shan Gao [1], Mingji Zong [1], Fengshou Liu [1], Qili Yang [1], Lanju Liang [1,*], Dawei Fang [2,*]

[1] School of Opto-electronic Engineering, Zaozhuang University, Zaozhuang 277160, China

[2] Institute of rare and scattered elements, College of chemistry, Liaoning University, Shenyang 110036, China

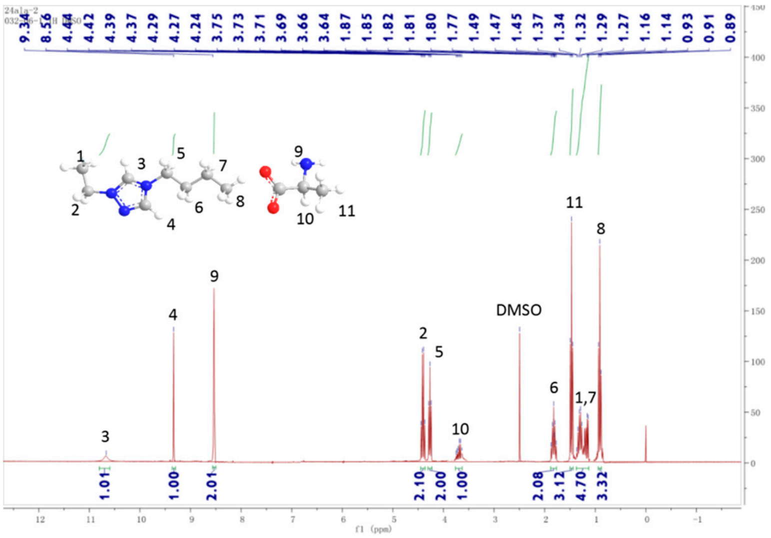

Figure S1. ^{1}H NMR spectroscopy of [Taz(2,4)][Ala] in DMSO.

E-mail address: lianglanju123@163.com (L. Liang); dwfang@lnu.edu.cn (D. Fang).

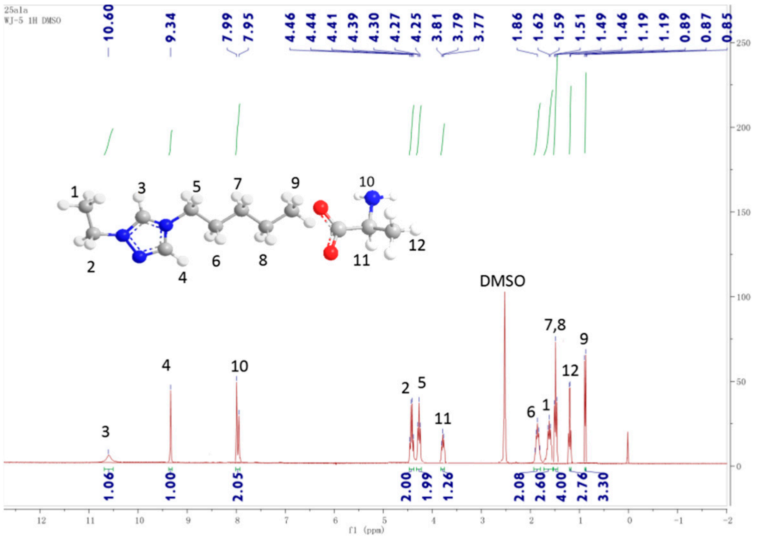

Figure S2. ^{1}H NMR spectroscopy of [Taz(2,5)][Ala] in DMSO.

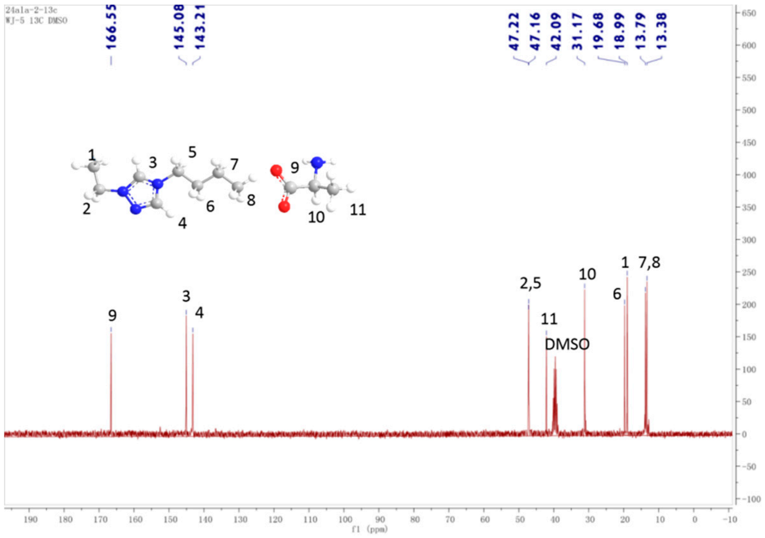

Figure S3. ^{13}C NMR spectroscopy of [Taz(2,4)][Ala] in DMSO.

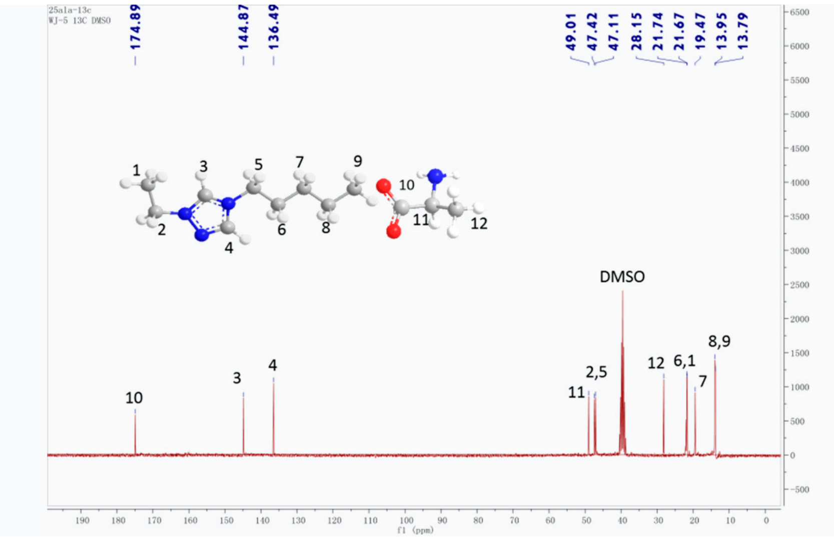

Figure S4. ^{13}C NMR spectroscopy of [Taz(2,5)][Ala] in DMSO.

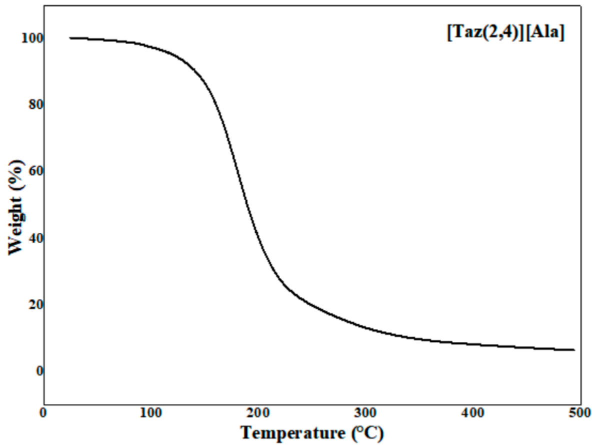

Figure S5. Thermogravimetry of [Taz(2,4)][Ala].

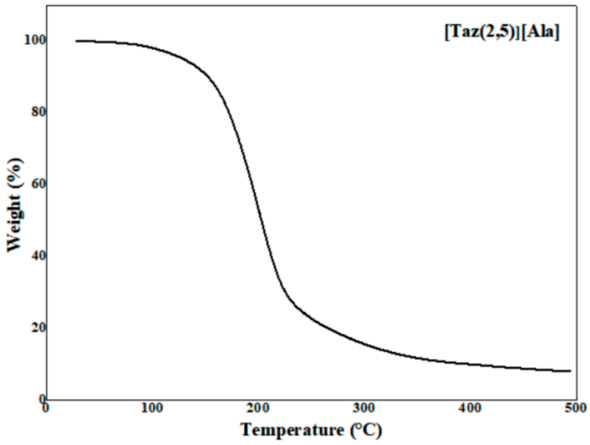

Figure S6. Thermogravimetry of [Taz(2,5)][Ala].

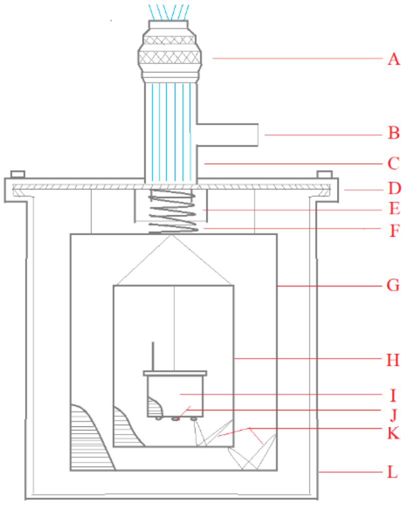

Figure S7. Cross-sectional diagram of adiabatic calorimetric cryostat: A is the sealing junction unit; B is the high vacuum system; C is the vacuum tube; D is the fuse gasket; E is the wire temperature control ring; F is the wire bundle; G and H are respectively the outer and inner adiabatic shields; I is the sample cell; J is the miniature platinum resistance thermometer; K are the chromel–copper thermocouples; L is a vacuum can.

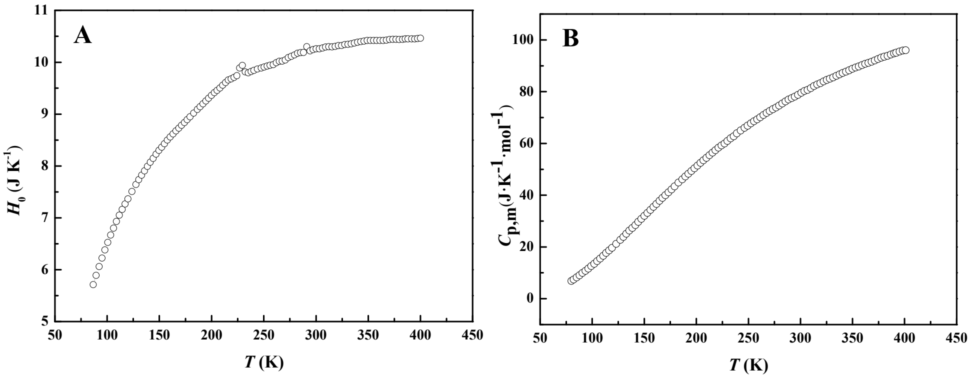

Figure S8. The heat capacities of empty equivalent (A) and α-Al_2O_3 (B) in the temperature range from 78 K to 400 K.

Table S1. Density, ρ (kg·cm^{-3}), values of [Taz(2,4)][Ala] and [Taz(2,5)][Ala] containing various mass fraction of water, w_2, at T = (288.15 - 318.15) K.

[Taz(2,4)][Ala]								
T / K	10^3w_2 = 5.12	10^3w_2 = 8.31	10^3w_2 = 10.74	10^3w_2 = 13.05	10^3w_2 = 15.37	10^3w_2 = 0	r^2	sd ×10^{-2}
288.15	1083.172	1083.021	1082.925	1082.799	1082.697	1083.41	0.998	1.06
293.15	1079.719	1079.564	1079.474	1079.331	1079.257	1079.95	0.991	2.12
298.15	1076.409	1076.245	1076.157	1076.051	1075.947	1076.63	0.997	1.26
303.15	1072.625	1072.476	1072.362	1072.269	1072.176	1072.84	0.998	1.02
308.15	1069.470	1069.345	1069.258	1069.159	1069.090	1069.66	0.997	0.98
313.15	1065.672	1065.530	1065.441	1065.348	1065.232	1065.89	0.995	1.55
318.15	1061.902	1061.729	1061.674	1061.559	1061.472	1062.11	0.995	1.51
[Taz(2,5)][Ala]								
T / K	10^3w_2 = 5.26	10^3w_2 = 8.03	10^3w_2 = 10.45	10^3w_2 = 12.98	10^3w_2 = 15.49	10^3w_2 = 0	r^2	sd ×10^{-3}
288.15	1061.010	1060.891	1060.777	1060.667	1060.547	1061.25	0.999	5.15
293.15	1057.487	1057.363	1057.249	1057.146	1057.027	1057.72	0.999	5.90
298.15	1053.963	1053.836	1053.729	1053.610	1053.507	1054.20	0.999	5.61
303.15	1050.435	1050.311	1050.204	1050.091	1049.986	1050.66	0.999	3.43
308.15	1046.833	1046.733	1046.645	1046.550	1046.460	1047.03	0.999	1.61
313.15	1043.320	1043.201	1043.096	1042.995	1042.885	1043.54	0.999	3.62
318.15	1039.815	1039.699	1039.597	1039.495	1039.385	1040.04	0.999	2.93

Standard uncertainties u are $u(T)$ = 0.01 K, $u(p)$ = 0.002 MPa, $u(w_2)$ = 0.0001, and the expanded uncertainty $U(\rho)$ = 0.1 Kg·m^{-3} with 0.95 level of confidence (k = 2); w_2 is the various mass fraction of water; r^2 is the correlation coefficient square and sd is the standard deviation.

Table S2. Surface tension, γ ($N \cdot m^{-1}$) values of [Taz(2,4)][Ala] and [Taz(2,5)][Ala] containing various mass fraction of water, w_2, at T = (288.15 - 318.15) K.

[Taz(2,4)][Ala]								
T / K	10^3w_2 = 5.12	10^3w_2 = 8.31	10^3w_2 = 10.74	10^3w_2 = 13.05	10^3w_2 = 15.37	10^3w_2 = 0	r^2	sd × 10^{-5}
288.15	0.0448	0.0451	0.0454	0.0457	0.0461	0.0441	0.997	2.84
293.15	0.0444	0.0447	0.0450	0.0453	0.0458	0.0437	0.994	5.93
298.15	0.0441	0.0444	0.0447	0.0450	0.0455	0.0434	0.994	5.93
303.15	0.0438	0.0441	0.0444	0.0447	0.0452	0.0431	0.994	5.93
308.15	0.0434	0.0437	0.0440	0.0443	0.0448	0.0427	0.994	5.93
313.15	0.0430	0.0433	0.0436	0.0439	0.0444	0.0423	0.994	5.93
318.15	0.0426	0.0429	0.0432	0.0435	0.0440	0.0419	0.994	5.93
[Taz(2,5)][Ala]								
T / K	10^3w_2 = 5.26	10^3w_2 = 8.03	10^3w_2 = 10.45	10^3w_2 = 12.98	10^3w_2 = 15.49	10^3w_2 = 0	r^2	sd × 10^{-5}
288.15	0.0412	0.0415	0.0418	0.0421	0.0423	0.0406	0.994	4.75
293.15	0.0409	0.0412	0.0415	0.0418	0.0420	0.0403	0.994	4.75
298.15	0.0406	0.0409	0.0412	0.0415	0.0417	0.0400	0.994	4.75
303.15	0.0403	0.0406	0.0409	0.0412	0.0414	0.0397	0.994	4.75
308.15	0.0399	0.0402	0.0405	0.0408	0.0410	0.0393	0.994	4.75
313.15	0.0395	0.0398	0.0401	0.0404	0.0406	0.0389	0.994	4.75
318.15	0.0391	0.0394	0.0397	0.0400	0.0402	0.0385	0.994	4.75

Standard uncertainties u are $u(T)$ = 0.01 K, $u(p)$ = 0.002 MPa, $u(w_2)$ = 0.0001, and the expanded uncertainty $U(\gamma)$ = 0.0001 $N \cdot m^{-1}$ with 0.95 level of confidence (k = 2); w_2 is the various mass fraction of water; r^2 is the correlation coefficient square and sd is the standard deviation.

Table S3. The fitting coefficients of thermophysical properties dependence on temperature for [Taz(2,4)][Ala] and [Taz(2,5)][Ala].

Thermophysical properties	Fitting coefficients	[Taz(2,4)][Ala]	[Taz(2,5)][Ala]
Density (288.15 - 318.15 K)	a_0 / kg·m^{-3}	1287.276	1265.351
	a_1 / kg· K^{-1}·m^{-3}	-0.707	-0.708
	δ / kg·m^{-3}	0.007	0.001
	r^2	0.999	0.999
Surface tension (288.15 - 318.15 K)	10^2 b_0 / N·m^{-1}	6.490	6.083
	10^5 b_1 / N·K^{-1}·m^{-1}	-7.214	-7.000
	10^4 δ / N·m^{-1}	5.395	6.987
	r^2	0.997	0.994
Isobaric heat capacity (238 - 390 K)	c_0 / J·K^{-1}·mol^{-1}	2222.376	1102.671
	c_1 / J·K^{-2}·mol^{-1}	-17.480	-5.930
	10^2 c_2 / J·K^{-3}·mol^{-1}	5.665	1.909
	10^5 c_3 / J·K^{-4}·mol^{-1}	-5.681	-1.662
	10^5 δ/ J·K^{-1}·mol^{-1}	0.39	0.42
	r^2	0.995	0.998
Thermal conductivity (288.15 - 318.15 K)	d_0 / W·m^{-1}·K^{-1}	0.209	0.201
	10^5 d_1 / W·m^{-1}·K^{-2}	-6.643	-6.786
	10^4 δ / W·m^{-2}·K^{-1}	5.395	5.395
	r^2	0.996	0.996

$\delta = (\sum_{i=1}^{n}(C_{p,\mathrm{exp}} - C_{p,\mathrm{cal}})^2/(n-m))^{1/2}$ where δ is standard mean deviation from the regression line, n is the number of experimental points and m is the number of fitted coefficients; r^2 is the correlation coefficient square.

Table S4. The experimental molar heat capacities of [Taz(2,4)][Ala] in the temperature range of 78 to 390 K.

T / K	$C_{p,m}$/ J·K^{-1}·mol^{-1}	T / K	$C_{p,m}$/ J·K^{-1}·mol^{-1}	T / K	$C_{p,m}$/ J·K^{-1}·mol^{-1}
78.379	176.295	184.199	356.211	288.983	529.209
81.462	181.162	187.898	401.042	291.885	530.428
83.461	186.580	190.724	466.434	294.709	532.194
86.382	191.664	193.613	490.111	297.528	532.546
89.173	197.155	196.546	491.827	300.357	537.397
91.996	202.661	199.469	492.911	303.195	541.452
94.816	207.485	202.385	493.711	306.038	543.660
97.638	211.287	205.295	495.272	308.888	547.193
100.466	216.163	208.200	496.027	311.740	556.613
103.302	220.532	211.099	498.535	314.595	565.719
106.149	226.657	213.992	500.708	317.457	569.582
108.983	231.679	216.886	501.615	320.323	572.414
111.800	235.402	219.800	503.661	323.287	581.667
114.635	240.645	222.754	504.686	326.261	582.951
117.492	247.622	225.615	505.575	329.247	586.402
120.341	251.698	228.509	506.892	332.248	588.268
124.310	257.633	231.540	384.980	335.263	592.169
128.266	263.357	234.529	1104.321	338.302	595.411
131.105	267.407	237.350	3539.827	341.368	597.661
133.965	272.008	240.258	1487.193	344.561	598.545
136.849	275.494	243.819	501.126	347.741	604.896
139.757	277.408	247.333	504.651	350.990	607.671
142.639	282.362	250.200	505.735	354.428	612.712
145.502	286.029	253.056	506.303	357.994	616.027
148.391	287.862	255.903	507.045	361.759	620.346
151.307	293.740	258.656	509.595	364.537	625.663
154.205	299.460	261.403	511.031	367.012	630.739
157.087	301.930	264.137	512.573	370.145	631.362
160.000	305.417	266.862	514.366	372.472	634.475
162.944	308.363	269.567	515.754	375.257	635.721
165.869	313.149	272.277	517.157	377.935	639.872

168.779	316.033	275.006	521.037	380.129	645.061
171.677	318.488	277.718	523.938	383.831	645.477
174.563	323.008	280.431	525.936	385.749	647.552
177.485	330.106	283.251	526.294	388.016	652.430
180.443	336.860	286.141	528.846	390.211	656.436

The standard uncertainty (0.68 level of confidence): $u(T)$ = 0.001 K. $u(p)$ = 0.001MPa. The expanded uncertainties U_c (0.95 level of confidence): $U_c(C_{p,m})$ = 0.005 J·K^{-1}·mol^{-1}.

Table S5. The experimental molar heat capacities of [Taz(2,5)][Ala] in the temperature range of 79 to 400 K.

T / K	$C_{p,m}$/ J·K^{-1}·mol^{-1}	T / K	$C_{p,m}$/ J·K^{-1}·mol^{-1}	T / K	$C_{p,m}$/ J·K^{-1}·mol^{-1}
79.940	186.503	169.536	308.288	294.742	588.414
82.043	189.321	172.141	312.088	297.707	591.708
84.726	193.896	174.763	315.620	300.684	595.555
87.525	198.159	177.460	317.514	303.666	599.393
90.269	204.740	180.169	323.898	306.640	602.651
93.038	210.824	183.606	365.177	309.604	604.017
95.780	215.436	187.249	378.407	312.564	607.378
98.525	219.732	190.102	380.773	315.522	609.222
101.242	226.743	192.953	383.855	318.471	611.667
103.932	230.711	195.818	385.637	321.415	614.770
106.619	235.813	198.726	387.630	324.353	618.952
109.310	238.013	201.705	389.800	327.288	620.291
112.011	242.599	204.684	391.907	330.222	624.246
114.693	247.062	207.733	394.182	333.154	632.168
117.365	251.971	211.075	397.627	336.083	636.332
119.996	255.788	214.994	329.210	339.010	638.279
123.710	259.658	219.861	586.371	341.937	642.265
127.415	263.296	225.530	3922.850	344.864	647.360
130.037	267.195	232.090	710.204	347.791	648.082
132.720	272.717	238.910	550.288	350.718	651.244
135.414	276.538	246.245	552.096	353.646	658.743
138.074	278.864	252.553	555.007	356.577	665.217

140.702	282.955	257.610	557.107	359.510	668.547
143.349	287.043	262.170	559.245	362.446	673.716
146.015	288.154	266.205	561.583	365.389	676.138
148.656	289.318	269.913	563.405	368.337	675.013
151.275	292.746	273.412	567.868	371.295	676.981
153.876	292.844	276.700	572.017	374.261	682.322
156.453	294.106	279.755	574.304	377.242	688.959
159.057	297.240	282.799	576.489	380.240	693.862
161.687	301.408	285.779	579.042	383.260	700.516
164.312	302.314	288.810	581.874	386.427	703.864
166.930	304.718	291.790	585.390	390.003	706.956

The standard uncertainty (0.68 level of confidence): $u(T)$ = 0.001 K. $u(p)$ = 0.001MPa. The expanded uncertainties U_c (0.95 level of confidence): $U_c(C_{p,m})$ = 0.005 J·K^{-1}·mol^{-1}.

Table S6. The composition and operating temperature for most popularly used HTFs and ILs.

Heat-transfer liquids	Composition	Operating temperature
Therminol 66 [9]	hydrogenated terphenyl	273 - 618 K
Therminol VP-1 [10]	eutectic mixture of diphenyl and diphenyl ether	285 - 673 K
Therminol VP-3 [10]	no details are available	276 - 603 K
Marlotherm SH [10]	mixture of isomeric dibenzylbenzenes	268 - 623 K
Dowfrost [3]	propylene glycol, water	228 - 393 K
Dowtherm 4000 [3]	ethylene glycol, water	223 - 448 K
Syltherm 800 [11]	dimethyl polysiloxane	233 - 673 K
Fluorinert FC 70 [3]	perfluorocarbon	248 - 488 K
[C_2MIm][BF_4] [3]	1-ethyl-3-methylimidazolium, tetrafluoroborate	288 - 719 K
[C_2MIm][PF_6] [3]	1-ethyl-3-methylimidazolium, hexafluorophosphate	334 - > 673 K
[C_4MIm][PF_6] [3]	1-butyl-3-methylimidazolium, hexafluorophosphate	268 - > 673 K
[C_4MIm][TFSI] [11]	1-butyl-3-methylimidazolium, bis-(trifluoromethylsulfonyl)azanide	280 - > 673 K
[C_2MMIm][TFSI] [33]	1-ethyl-2,3-dimethyl-1H-imidazol-3-ium, bis-(trifluoromethylsulfonyl)azanide	293 - 766 K

[C_4MMIm][TFSI] [33]	1-butyl-2,3-dimethyl-1H-imidazol-3-ium, bis-(trifluoromethylsulfonyl)azanide	288 - 763 K
[C_3MPyr][TFSI] [8]	1-propyl-1-methylpyrrolidinium, bis-(trifluoromethylsulfonyl)imide	283 - 719 K
[C_4MPyr][TFSI] [8]	1-butyl-1-methylpyrrolidinium, bis-(trifluoromethylsulfonyl)imide	256 - 771 K
[Taz(2,4)][Acgly] [19]	1-ethyl-4-butyl-1,2,4-triazolium, *N*-Acetyl-Glycine	226 - 453 K
[Taz(2,4)][Acala] [19]	1-ethyl-4-butyl-1,2,4-triazolium, *N*-Acetyl-*L*-Alanine	229 - 454 K
[Taz(2,4)][Accys] [19]	1-ethyl-4-butyl-1,2,4-triazolium, *N*-Acetyl-*L*-Cysteine	229 - 452 K
[C_4Eim][SbF_6] [20]	1-butyl-3-ethylimidazolium, hexafluoride antimonite	257 - 660 K
[Taz(2,4)][Ala][a]	1-ethyl-4-butyl-1,2,4-triazolium, *L*- Alanine	238 - 447 K
[Taz(2,5)][Ala][a]	1-ethyl-4-amyl-1,2,4-triazolium, *L*- Alanine	226 - 451 K

[a] studied in this work.

Table S7. The thermal conductivity for [Taz(2,4)][Ala], [Taz(2,5)][Ala], most popularly used HTFs and widely studied ILs at 313.15 K.

ILs	λ / W·m^{-1}·K^{-1}	Commercial HTFs	λ / W·m^{-1}·K^{-1}	Commercial HTFs	λ / W·m^{-1}·K^{-1}
[C_2MIm][$N(CN)_2$] [40]	0.178	Therminol 66 [10]	0.117	Therminol 54[b]	0.126
[C_4MIm][$N(CN)_2$] [40]	0.163	Therminol VP-1 [10]	0.134	Therminol 55[b]	0.1261
[C_4MPyr][$N(CN)_2$] [40]	0.050	Therminol VP-3 [9]	0.115	Therminol 59[b]	0.1199
[C_4MIm][TFSI] [35]	0.114	Dowtherm A [1]	0.136	Therminol 62[b]	0.1212
[C_2MMIm][TFSI] [33]	0.1163	Dowtherm G [1]	0.124	Therminol 68[b]	0.122
[C_4MMIm][TFSI] [33]	0.1187	Dowtherm J [1]	0.124	Therminol 72[b]	0.137
[C_3MPyr][TFSI] [3]	0.1181	Dowtherm MX [1]	0.121	Therminol 75 (343.15 K)[b]	0.1314
[C_4MPyr][TFSI] [3]	0.1240	Dowtherm Q [1]	0.120	Therminol ADX-10[b]	0.1213
[C_2MIm][SCN] [1]	0.182	Dowtherm RP [1]	0.129	Therminol D-12[b]	0.1068

$[C_4MIm][SCN]$ [1]	0.169	Dowtherm T [1]	0.127	Therminol LT[b]	0.1207
$[C_2MIm][BF_4]$ (323 K) [1]	0.196	Syltherm XLT [1]	0.106	Therminol VLT[b]	0.0993
$[C_4MIm][PF_6]$ (323 K) [26]	0.146	Syltherm 800 [1]	0.131	Therminol SP[b]	0.1261
$[C_6MIm][PF_6]$ (323 K) [26]	0.146	Syltherm HF [1]	0.102	Therminol XP[b]	0.1141
$[C_2MIm][C(CN)_3]$ [1]	0.176	Paratherm HR [1]	0.116	Marlotherm LH[b]	0.129
$[C_2MIm][C_2H_5SO_4]$ [40]	0.171	Paratherm MR [1]	0.130	Marlotherm N[b]	0.134
$[P_{4,4,4.16}][N(CN)_2]$ [40]	0.161	Globaltherm Omniterm [1]	0.138	Paratherm LR (311.15 K)[b]	0.086
$[C_2MIm][CH_3SO_3]$ [36]	0.202	Globaltherm Syntec [1]	0.119		
$[C_2MIm][OAc]$ [9]	0.190	Marlotherm SH [1]	0.128		
[Taz(2,4)][Acala] [19]	0.1954				
$[C_4Eim][SbF_6]$ [20]	0.1574				
$[C_5Eim][SbF_6]$ [20]	0.1528				
[Taz(2,4)][Ala][a]	0.1880				
[Taz(2,5)][Ala][a]	0.1776				

[a] Studied in this work, [b] btained from the product information brochure available online and/or requested to the supplier.

Table S8. Values of the boiling temperature (T_b), critical temperature (T_c), and important parameters for the group contribution method proposed by Oster et al..

Parameters	[Taz(2,4)][Ala]	[Taz(2,5)][Ala]
$\sum_{j=1}^{k} n_j \Delta T_b$	575.05	597.93
T_b / K	773.25	796.13
$\sum_{j=1}^{k} n_j \Delta T_C$	0.2463	0.2632
T_c / K	1018.887	1037.424
$\sum_{j=1}^{k} n_j \Delta \lambda_{0,j}$	10.3742	11.0437

Table S9. The first (series 1), the second (series 2) and the third (series 3) experimental heat capacities of the empty equivalent in the temperature range of 78 to 400 K.

T / K	$C_{p,m}$/ J·K^{-1}·mol^{-1}	T / K	$C_{p,m}$/ J·K^{-1}·mol^{-1}	T / K	$C_{p,m}$/ J·K^{-1}·mol^{-1}
		Series 1			
78.830	5.200	186.324	9.090	294.228	10.219
80.978	5.319	189.045	9.144	297.153	10.245
83.884	5.519	191.788	9.198	300.183	10.260
86.682	5.713	194.513	9.253	303.233	10.261
89.473	5.891	197.188	9.307	306.233	10.274
92.263	6.061	199.899	9.362	309.258	10.293
95.054	6.225	202.629	9.414	312.265	10.301
97.849	6.383	205.290	9.458	315.294	10.298
100.650	6.528	207.973	9.504	318.351	10.306
103.426	6.670	210.685	9.558	321.423	10.321
106.182	6.802	213.345	9.610	324.504	10.323
108.935	6.932	216.029	9.658	327.587	10.341
111.667	7.053	218.747	9.681	330.650	10.346

114.385	7.164	221.450	9.687	333.705	10.354
117.125	7.266	224.187	9.782	336.761	10.372
119.855	7.367	226.874	9.869	339.818	10.387
123.706	7.509	229.471	9.941	342.877	10.397
127.546	7.643	232.219	9.814	345.934	10.408
130.280	7.737	235.076	9.795	348.992	10.418
133.039	7.824	237.856	9.820	352.048	10.421
135.767	7.906	240.500	9.842	355.103	10.420
138.525	7.992	243.880	9.870	358.163	10.420
141.308	8.071	247.537	9.889	361.232	10.419
144.069	8.147	250.350	9.908	364.305	10.419
146.806	8.228	253.179	9.927	367.381	10.426
149.520	8.300	256.028	9.942	370.464	10.437
152.213	8.363	259.032	9.954	373.549	10.441
154.937	8.428	261.979	9.990	376.639	10.440
157.703	8.499	264.812	10.019	379.738	10.439
160.455	8.558	267.669	10.021	382.841	10.442
163.182	8.618	270.539	10.044	385.458	10.450
165.900	8.677	273.548	10.090	388.025	10.451
168.613	8.730	276.406	10.113	391.672	10.450
171.313	8.785	279.357	10.140	394.571	10.449
174.000	8.839	282.354	10.174	397.269	10.456
176.677	8.892	285.203	10.187	400.013	10.463
179.404	8.947	288.108	10.190		
182.873	9.018	291.146	10.300		
		Series 2			
79.747	5.246	182.151	9.003	291.183	10.300
80.964	5.318	186.368	9.091	297.190	10.245
83.965	5.525	189.069	9.145	300.176	10.260

86.765	5.718	191.804	9.199	303.253	10.261
89.556	5.897	194.561	9.254	306.235	10.274
92.341	6.065	197.241	9.308	309.272	10.293
95.126	6.229	199.911	9.362	312.279	10.301
97.915	6.386	202.680	9.415	315.291	10.298
100.709	6.531	205.350	9.459	318.342	10.306
103.513	6.674	208.002	9.505	321.410	10.321
106.256	6.806	210.737	9.559	324.488	10.323
108.994	6.935	213.412	9.612	327.576	10.341
111.748	7.057	216.058	9.658	330.657	10.346
114.449	7.167	218.803	9.681	333.707	10.354
117.175	7.268	221.484	9.687	336.769	10.372
119.922	7.370	224.228	9.784	339.826	10.387
122.626	7.470	226.954	9.870	342.884	10.397
127.642	7.646	229.632	9.959	345.947	10.408
130.330	7.738	232.220	9.814	349.004	10.418
133.103	7.826	235.063	9.795	352.065	10.421
135.845	7.909	237.922	9.820	355.119	10.420
138.556	7.993	240.627	9.843	358.178	10.420
141.351	8.072	243.207	9.866	361.244	10.419
144.121	8.149	247.577	9.889	364.317	10.419
146.866	8.230	250.374	9.908	367.393	10.426
149.589	8.302	253.204	9.928	370.473	10.437
152.291	8.364	256.040	9.942	373.559	10.441
154.970	8.428	259.048	9.954	376.648	10.440
157.740	8.500	262.019	9.990	379.743	10.439
160.499	8.559	264.834	10.020	382.848	10.442
163.236	8.619	267.681	10.021	385.167	10.449
165.952	8.678	270.557	10.045	388.492	10.450

168.667	8.731	273.582	10.090	391.764	10.450
171.377	8.786	276.420	10.113	394.267	10.449
174.067	8.840	279.386	10.140	397.264	10.456
176.747	8.893	282.412	10.175	400.576	10.463
179.414	8.947	285.221	10.187		
		Series 3			
79.456	5.231	185.137	9.066	289.634	10.194
82.535	5.423	187.822	9.120	292.651	10.408
85.346	5.622	190.564	9.174	295.700	10.231
88.142	5.807	193.314	9.229	298.658	10.255
90.934	5.981	196.008	9.283	301.734	10.261
93.728	6.146	198.679	9.337	304.755	10.265
96.525	6.310	201.429	9.392	307.774	10.285
99.327	6.460	204.121	9.440	310.788	10.299
102.134	6.604	206.774	9.483	313.795	10.300
104.883	6.741	209.486	9.533	316.841	10.300
107.652	6.872	212.177	9.588	319.902	10.314
110.419	6.999	214.822	9.638	322.980	10.322
113.129	7.114	217.544	9.675	326.063	10.332
115.863	7.220	220.235	9.682	329.142	10.345
118.618	7.321	222.960	9.724	332.187	10.349
121.330	7.423	225.688	9.845	335.245	10.363
126.330	7.601	228.336	9.932	338.297	10.381
128.994	7.693	230.852	9.838	341.353	10.392
131.773	7.785	233.761	9.795	344.413	10.403
134.518	7.868	236.633	9.808	347.463	10.414
137.233	7.952	239.334	9.831	350.522	10.421
140.037	8.037	241.916	9.856	353.574	10.421
142.809	8.110	246.097	9.880	356.630	10.420

145.559	8.192	249.039	9.899	359.690	10.420
148.288	8.269	251.870	9.918	362.764	10.419
150.994	8.335	254.697	9.937	365.835	10.421
153.678	8.396	257.559	9.947	368.911	10.431
156.446	8.468	260.558	9.969	371.997	10.440
159.210	8.532	263.483	10.010	375.082	10.441
161.954	8.590	266.332	10.022	378.173	10.439
164.672	8.651	269.200	10.027	381.276	10.440
167.390	8.706	272.063	10.069	384.378	10.447
170.102	8.760	275.057	10.104	387.246	10.451
172.792	8.815	277.936	10.124	391.042	10.450
175.476	8.868	280.871	10.158	394.346	10.449
178.150	8.921	283.839	10.184	397.894	10.458
180.937	8.978	286.729	10.188	400.243	10.463

The standard uncertainty (0.68 level of confidence): $u(T)$ = 0.001 K. $u(p)$ = 0.001MPa. The expanded uncertainties U_c (0.95 level of confidence): $U_c(C_{p,m})$ = 0.005 J·K^{-1}·mol^{-1}.

Table S10. The heat capacities of α-Al_2O_3 in the temperature range of 78 to 400 K.

T / K	$C_{p,m}$ / J·K^{-1}·mol^{-1}	T / K	$C_{p,m}$ / J·K^{-1}·mol^{-1}	T / K	$C_{p,m}$ / J·K^{-1}·mol^{-1}
79.904	6.862	186.546	46.099	294.633	78.086
82.062	7.424	189.456	47.207	297.704	78.764
85.066	8.238	192.359	48.277	300.832	79.569
87.839	9.049	195.257	49.306	303.825	80.252
90.657	9.910	198.152	50.374	306.858	80.712
93.482	10.747	201.048	51.448	309.922	81.372
96.279	11.635	203.945	52.505	313.017	82.184
99.088	12.568	206.839	53.465	316.092	82.802
101.914	13.481	209.723	54.417	319.179	83.321
104.761	14.504	212.614	55.438	322.233	83.957

107.628	15.520	215.494	56.428	325.266	84.552
110.483	16.559	218.343	57.370	328.317	85.021
113.327	17.681	221.239	58.331	331.367	85.519
116.200	18.658	224.167	59.232	334.451	86.095
119.070	19.649	227.022	59.974	337.531	86.718
123.064	21.185	229.863	60.944	340.609	87.252
127.039	22.753	232.866	61.980	343.684	87.714
129.893	23.874	235.931	62.739	346.762	88.273
132.777	25.084	238.922	63.916	349.845	88.871
135.633	26.333	242.582	65.014	352.926	89.348
138.520	27.351	246.307	66.058	356.012	89.763
141.438	28.451	249.311	66.862	359.093	90.257
144.335	29.682	252.406	67.742	362.171	90.768
147.211	30.854	255.425	68.599	365.245	91.177
150.066	31.991	258.412	69.330	368.307	91.582
152.958	33.121	261.423	70.102	371.365	92.065
155.884	34.280	264.435	70.971	374.425	92.640
158.806	35.445	267.453	71.782	377.495	93.163
161.704	36.603	270.475	72.539	380.573	93.528
164.582	37.814	273.500	73.226	383.659	93.865
167.446	38.933	276.535	73.887	386.746	94.364
170.298	39.993	279.578	74.628	389.846	94.811
173.192	41.128	282.598	75.411	392.078	95.088
176.128	42.232	285.596	76.220	395.183	95.502
179.049	43.364	288.604	76.968	398.215	95.878
182.798	44.879	291.644	77.542	400.997	96.015

The standard uncertainty (0.68 level of confidence): $u(T)$ = 0.001 K. $u(p)$ = 0.001MPa. The expanded uncertainties U_c (0.95 level of confidence): $U_c(C_{p,m})$ = 0.005 J·K^{-1}·mol^{-1}.

Table S11. The first experimental data, fitted values, and relative deviation of the empty equivalent in the temperature range of 78 to 400 K.

T / K	$H_{0(exp)}$ J·K^{-1}	$H_{0(fit)}$ J·K^{-1}	A/ %	T / K	$H_{0(exp)}$ J·K^{-1}	$H_{0(fit)}$ J·K^{-1}	A/ %
78.830	5.200	5.231	0.361	237.856	9.820	9.821	-0.016
80.978	5.319	5.300	0.354	240.500	9.842	9.841	0.007
83.884	5.519	5.516	0.047	243.880	9.870	9.866	0.041
86.682	5.713	5.711	0.025	247.537	9.889	9.891	-0.025
89.473	5.891	5.894	-0.042	250.350	9.908	9.909	-0.015
92.263	6.061	6.066	-0.088	253.179	9.927	9.926	0.009
95.054	6.225	6.229	-0.056	256.028	9.942	9.944	-0.017
97.849	6.383	6.383	-0.005	259.032	9.954	9.963	-0.089
100.650	6.528	6.530	-0.029	261.979	9.990	9.983	0.072
103.426	6.670	6.669	0.014	264.812	10.019	10.004	0.150
106.182	6.802	6.800	0.037	267.669	10.021	10.028	-0.069
108.935	6.932	6.925	0.105	270.539	10.044	10.054	-0.104
111.667	7.053	7.044	0.131	273.548	10.090	10.085	0.054
114.385	7.164	7.158	0.091	276.406	10.113	10.114	-0.010
117.125	7.266	7.268	-0.026	279.357	10.140	10.143	-0.034
119.855	7.367	7.373	-0.074	282.354	10.174	10.169	0.046
123.706	7.509	7.514	-0.070	285.203	10.187	10.186	0.005
127.546	7.643	7.648	-0.068	288.108	10.190	10.191	-0.013
130.280	7.737	7.739	-0.028	294.228	10.219	10.220	-0.012
133.039	7.824	7.827	-0.042	297.153	10.245	10.242	0.029
135.767	7.906	7.911	-0.065	300.183	10.260	10.258	0.021
138.525	7.992	7.993	-0.012	303.233	10.261	10.269	-0.081
141.308	8.071	8.073	-0.030	306.233	10.274	10.278	-0.039
144.069	8.147	8.150	-0.031	309.258	10.293	10.286	0.073
146.806	8.228	8.223	0.067	312.265	10.301	10.293	0.080
149.520	8.300	8.293	0.087	315.294	10.298	10.300	-0.023

152.213	8.363	8.360	0.028	318.351	10.306	10.309	-0.027
154.937	8.428	8.426	0.017	321.423	10.321	10.318	0.028
157.703	8.499	8.491	0.095	324.504	10.323	10.328	-0.052
160.455	8.558	8.554	0.042	327.587	10.341	10.339	0.017
163.182	8.618	8.615	0.033	330.650	10.346	10.350	-0.043
165.900	8.677	8.674	0.037	333.705	10.354	10.362	-0.075
168.613	8.730	8.732	-0.023	336.761	10.372	10.373	-0.008
171.313	8.785	8.788	-0.039	339.818	10.387	10.383	0.034
174.000	8.839	8.844	-0.056	342.877	10.397	10.393	0.036
176.677	8.892	8.898	-0.071	345.934	10.408	10.402	0.058
179.404	8.947	8.953	-0.075	348.992	10.418	10.410	0.082
182.873	9.018	9.022	-0.045	352.048	10.421	10.416	0.050
186.324	9.090	9.091	-0.009	355.103	10.420	10.421	-0.008
189.045	9.144	9.144	-0.003	358.163	10.420	10.425	-0.046
191.788	9.198	9.198	0.000	361.232	10.419	10.428	-0.083
194.513	9.253	9.252	0.018	364.305	10.419	10.430	-0.103
197.188	9.307	9.304	0.031	367.381	10.426	10.431	-0.052
199.899	9.362	9.356	0.064	370.464	10.437	10.433	0.040
202.629	9.414	9.409	0.060	373.549	10.441	10.434	0.065
205.290	9.458	9.459	-0.006	376.639	10.440	10.436	0.037
207.973	9.504	9.508	-0.046	379.738	10.439	10.439	0.004
210.685	9.558	9.557	0.007	382.841	10.442	10.442	0.002
213.345	9.610	9.603	0.075	385.458	10.450	10.445	0.048
216.029	9.658	9.647	0.110	388.025	10.451	10.448	0.024
218.747	9.681	9.688	-0.071	391.672	10.450	10.453	-0.033
221.450	9.687	9.705	-0.291	394.571	10.449	10.457	-0.074
224.187	9.782	9.756	0.259	397.269	10.456	10.458	-0.022
235.076	9.795	9.801	-0.064	400.013	10.463	10.457	0.053

Table S12. The heat capacities of α-Al2O3 in the temperature range of 79 to 400 K.

T / K	$H_{0(exp)}$ /J·K^{-1}	$H_{0(fit)}$ /J·K^{-1}	A/ %	T / K	$H_{0(exp)}$ /J·K^{-1}	$H_{0(fit)}$ //J·K^{-1}	A/ %
79.904	6.862	6.874	-0.180	238.922	63.916	63.808	0.170
82.062	7.424	7.432	-0.098	242.582	65.014	64.889	0.193
85.066	8.238	8.246	-0.096	246.307	66.058	65.969	0.135
87.839	9.049	9.035	0.151	249.311	66.862	66.825	0.056
90.657	9.910	9.872	0.288	252.406	67.742	67.692	0.073
93.482	10.747	10.742	0.043	255.425	68.599	68.525	0.108
96.279	11.635	11.634	0.005	258.412	69.330	69.337	-0.010
99.088	12.568	12.558	0.080	261.423	70.102	70.141	-0.056
101.914	13.481	13.512	-0.232	264.435	70.971	70.934	0.052
104.761	14.504	14.498	0.042	267.453	71.782	71.715	0.094
107.628	15.520	15.513	0.048	270.475	72.539	72.484	0.075
110.483	16.559	16.543	0.099	273.500	73.226	73.242	-0.022
113.327	17.681	17.587	0.235	276.535	73.887	73.990	-0.140
116.200	18.658	18.658	-0.002	279.578	74.628	74.728	-0.134
119.070	19.649	19.743	-0.276	282.598	75.411	75.448	-0.049
123.064	21.185	21.274	-0.216	285.596	76.220	76.152	0.090
127.039	22.753	22.817	-0.281	288.604	76.968	76.846	0.159
129.893	23.874	23.936	-0.259	291.644	77.542	77.536	0.007
132.777	25.084	25.074	0.042	294.633	78.086	78.204	-0.151
135.633	26.333	26.206	0.286	297.704	78.764	78.879	-0.146
138.520	27.351	27.355	-0.013	300.832	79.569	79.555	0.017
141.438	28.451	28.519	-0.238	303.825	80.252	80.192	0.075
144.335	29.682	29.677	0.017	306.858	80.712	80.826	-0.141
147.211	30.854	30.827	0.086	309.922	81.372	81.456	-0.104
150.066	31.991	31.969	0.069	313.017	82.184	82.083	0.123
152.958	33.121	33.124	-0.010	316.092	82.802	82.695	0.129
155.884	34.280	34.291	-0.032	319.179	83.321	83.300	0.026

158.806	35.445	35.452	-0.021	322.233	83.957	83.888	0.082
161.704	36.603	36.600	0.007	325.266	84.552	84.463	0.105
164.582	37.814	37.736	0.207	328.317	85.021	85.032	-0.013
167.446	38.933	38.860	0.187	331.367	85.519	85.592	-0.086
170.298	39.993	39.973	0.049	334.451	86.095	86.149	-0.063
173.192	41.128	41.097	0.076	337.531	86.718	86.697	0.024
176.128	42.232	42.228	0.010	340.609	87.252	87.235	0.019
179.049	43.364	43.345	0.043	343.684	87.714	87.764	-0.057
182.798	44.879	44.766	0.252	346.762	88.273	88.285	-0.014
186.546	46.099	46.171	-0.156	349.845	88.871	88.799	0.081
189.456	47.207	47.251	-0.092	352.926	89.348	89.304	0.050
192.359	48.277	48.317	-0.082	356.012	89.763	89.801	-0.043
195.257	49.306	49.371	-0.132	359.093	90.257	90.290	-0.037
198.152	50.374	50.413	-0.077	362.171	90.768	90.770	-0.003
201.048	51.448	51.444	0.008	365.245	91.177	91.242	-0.071
203.945	52.505	52.463	0.079	368.307	91.582	91.705	-0.134
206.839	53.465	53.471	-0.010	371.365	92.065	92.159	-0.102
209.723	54.417	54.462	-0.083	374.425	92.640	92.606	0.037
212.614	55.438	55.444	-0.011	377.495	93.163	93.047	0.125
215.494	56.428	56.410	0.032	380.573	93.528	93.481	0.050
218.343	57.370	57.354	0.028	383.659	93.865	93.910	-0.048
221.239	58.331	58.300	0.052	386.746	94.364	94.331	0.035
224.167	59.232	59.245	-0.022	389.846	94.811	94.746	0.068
227.022	59.974	60.153	-0.298	392.078	95.088	95.041	0.050
229.863	60.944	61.045	-0.166	395.183	95.502	95.444	0.061
232.866	61.980	61.975	0.008	398.215	95.878	95.831	0.049
235.931	62.739	62.909	-0.271	400.997	96.015	96.180	-0.171

Table S13. The heat capacities of α-Al_2O_3 in the temperature range of 80 to 400 K.

T / K	$H_{0(exp)}$ /J·K^{-1}	$H_{0(rec)}$ /J·K^{-1}	A/ %	T / K	$H_{0(exp)}$ /J·K^{-1}	$H_{0(rec)}$ /J·K^{-1}	A/ %
80	6.887	6.901	-0.204	245	65.693	65.649	0.067
85	8.220	8.234	-0.176	250	67.052	67.076	-0.036
90	9.711	9.678	0.342	255	68.485	68.466	0.028
95	11.219	11.221	-0.018	260	69.723	69.82	-0.139
100	12.859	12.855	0.031	265	71.129	71.137	-0.011
105	14.590	14.567	0.158	270	72.424	72.419	0.007
110	16.374	16.347	0.165	275	73.548	73.656	-0.147
115	18.243	18.184	0.324	280	74.735	74.871	-0.182
120	19.993	20.069	-0.379	285	76.060	76.053	0.009
125	21.951	21.993	-0.191	290	77.248	77.204	0.057
130	23.917	23.951	-0.142	295	78.159	78.324	-0.211
135	26.033	25.935	0.378	300	79.352	79.415	-0.079
140	27.886	27.935	-0.175	305	80.438	80.476	-0.047
145	29.959	29.943	0.053	310	81.392	81.509	-0.144
150	31.965	31.952	0.041	315	82.607	82.514	0.113
155	33.928	33.957	-0.085	320	83.482	83.493	-0.013
160	35.917	35.953	-0.100	325	84.506	84.445	0.072
165	37.985	37.934	0.134	330	85.287	85.373	-0.101
170	39.880	39.896	-0.040	335	86.206	86.276	-0.081
175	41.812	41.836	-0.057	340	87.156	87.155	0.001
180	43.759	43.752	0.016	345	87.939	88.011	-0.082
185	45.596	45.64	-0.096	350	88.898	88.844	0.061
190	47.415	47.499	-0.177	355	89.623	89.656	-0.037
195	49.213	49.326	-0.229	360	90.413	90.447	-0.038
200	51.059	51.121	-0.121	365	91.146	91.217	-0.078
205	52.864	52.881	-0.032	370	91.836	91.967	-0.142
210	54.513	54.606	-0.170	375	92.747	92.698	0.053

215	56.262	56.295	-0.059	380	93.469	93.411	0.062
220	57.920	57.948	-0.048	385	94.071	94.105	-0.036
225	59.448	59.564	-0.195	390	94.831	94.782	0.052
230	60.996	61.141	-0.237	395	95.477	95.441	0.038
235	62.481	62.682	-0.321	400	96.001	96.084	-0.086
240	64.288	64.184	0.162				
